# Infants’ Preference for Native Audiovisual Speech Dissociated from Congruency Preference

**DOI:** 10.1371/journal.pone.0126059

**Published:** 2015-04-30

**Authors:** Kathleen Shaw, Martijn Baart, Nicole Depowski, Heather Bortfeld

**Affiliations:** 1 Department of Psychology, University of Connecticut, Storrs, CT, United States of America; 2 BCBL. Basque Center on Cognition, Brain and Language, Donostia - San Sebastián, Spain; 3 Haskins Laboratories, New Haven, CT, United States of America; Max Planck Institute for Human Cognitive and Brain Sciences, GERMANY

## Abstract

Although infant speech perception in often studied in isolated modalities, infants' experience with speech is largely multimodal (i.e., speech sounds they hear are accompanied by articulating faces). Across two experiments, we tested infants’ sensitivity to the relationship between the auditory and visual components of audiovisual speech in their native (English) and non-native (Spanish) language. In Experiment 1, infants’ looking times were measured during a preferential looking task in which they saw two simultaneous visual speech streams articulating a story, one in English and the other in Spanish, while they heard either the English or the Spanish version of the story. In Experiment 2, looking times from another group of infants were measured as they watched single displays of congruent and incongruent combinations of English and Spanish audio and visual speech streams. Findings demonstrated an age-related increase in looking towards the native relative to non-native visual speech stream when accompanied by the corresponding (native) auditory speech. This increase in native language preference did not appear to be driven by a difference in preference for native vs. non-native audiovisual congruence as we observed no difference in looking times at the audiovisual streams in Experiment 2.

## Introduction

Infants' experience with speech is largely multimodal as the speech sounds they hear are accompanied by articulating faces. Yet, infant speech perception is often studied in isolated modalities [1, 2] and characterized by a process commonly referred to as perceptual narrowing [2–5]. Perceptual narrowing reflects a gradual loss in infants' ability to discriminate or process rarely-encountered stimuli and has been documented in language domains including biological speech [6] and linguistic signs [7]. By 6 months of age, infants are better able to discriminate vowel contrasts found in their own language than vowel contrasts in non-native languages [3] and at around 9 months of age, infants lose sensitivity for consonantal non-native speech contrasts [4], and non-native phonemes that resemble phonemes in the native language are likely to lose discriminability as well [5]. Perceptual narrowing seems to extend to visual speech, as demonstrated by Weikum et al. [2] who exposed 4-, 6-, and 8-month-old monolingual English infants to either English or French silent faces producing sentences, and found that 4- and 6-month-old, but not 8-month-old, infants detected the difference between English and French visual speech. In contrast, 8-month-old bilingual (English-French) infants were able to detect the difference between the two languages, presumably as a result of their enhanced language experience [2].

As has already been alluded to, however, speech perception is generally not an auditory-only process (let alone a visual-only one) but an audiovisual (henceforth, AV) one, and may even have properties that are not tied to a specific modality (i.e., they are amodal). Recently, Lewkowicz and Pons [8] used unimodal speech to show that amodal representations for native speech emerge at around 10 to 12 months of age. They familiarized infants to native (English) or non-native (Spanish) fluent (i.e., continuous) auditory speech, after which two simultaneous silent visual speech videos were presented, one articulating the previously heard speech segment, the other articulating the same segment in the other language. The key finding was that 10- to 12-month-olds looked less at the native visual speech stream when previously familiarized to native auditory speech, most likely because they had detected the correspondence between sight and sound and preferred the novel visual speech. However, a very similar experiment by Kubicek and co-workers [9] revealed similar effects occurring already at 6 months of age. One important difference between both studies that may explain the considerable age difference at which the effects were observed is that in [9], the unimodally presented speech signals were from the same speaker and the same recording, whereas in [8], the auditory speech and visual speech were never from the same speaker. In other words, apart from the fact that unimodal speech segments were presented sequentially, the correlation between what was heard and seen was perfect in [9], but not in [8].

The importance of the correlation between sight and sound was underscored in a second experiment reported by Kubicek et al [9] in which auditory and visual speech were presented simultaneously. The authors presented fluent auditory speech in the native or non-native language together with two silent videos of a bilingual speaker simultaneously articulating the segment in both languages, such that only one video matched the sound. Interestingly, 6-month-olds no longer showed a native language preference and matched the auditory with visual speech in both languages and the authors concluded that infants may effectively rely on purely temporal information to detect the correspondence between an articulating face and a speech sound (see [10] for similar arguments based on results obtained with artificial speech). However, 12-month-olds only matched non-native speech [9], which was accounted for with the argument that the non-native nature of the language had differentially modulated looking behavior because at that age, the correlation between the moving mouth and the auditory speech signal loses importance for native speech (instead, infants look more at the eyes), while still being crucial for non-native speech [9, 11, 12]. Although this interpretation is conceivable, Kubicek et al [9] had presented 60 seconds of visual-only familiarization (with both visual languages presented simultaneously, side by side) before the audiovisual (henceforth AV) stimuli, which may have contributed to (but cannot fully explain) the non-native preference shown by the 12-month-olds. This is because there was a slight preference for the native visual speech during silent familiarization, implying that upon re-appearance, the native visual speech was simply less interesting to the infants as compared to the non-native visual speech.

Indeed, as the perceptual system becomes more tuned to the native language in the second half of the first year of life [12, 13], it seems likely that infants' preference for native over non-native AV speech increases during that time, which in turn is likely to be reflected by an increase in looking at native relative to non-native AV speech. It is well established that AV correspondence detection is characterized by longer looking times towards visual displays that match a simultaneously presented sound. For instance, Kuhl and Meltzoff [14] observed that when hearing /a/ vowels, 4-month-old infants looked longer at a face articulating /a/ than at a simultaneously articulating face producing /i/, a findings that was later replicated and also found for infants as young as 2 months of age [15, 16]. Similarly, 8-month-olds prefer to look at a gender congruent visual display relative to an incongruent one when hearing a gender specific voice [17], 5- to 15-month-olds look longer at a visual speech stream that matches a three syllable non-word with degraded phonetic detail [10], and 4- and 6-month-olds even prefer to look at a cooing monkey face over a monkey face producing a grunt if they are simultaneously hearing the cooing sound [18].

The goal of the current study was to determine whether infants' preference for native AV speech increases during the second half of the first year of life. We tested this via a preferential looking paradigm in which we presented fluent speech to English learning infants without using familiarization (and without consecutive presentations of the same stimulus, which is the default in familiarization–test procedures). Infants heard short English (their native language) or Spanish (a non-native language) stories while they saw two simultaneous visual speech streams articulating the story, one in English and the other in Spanish. To exclude any differential influence of the speakers’ eyes on looking at the native/non-native speech streams [12] and to emphasize the correlation between the moving mouth and the auditory speech sounds, stimuli were videos cropped to show only the lower half of the face. These were presented to infants who ranged from 5 to 10 months of age. The wide age range was to ensure that we included infants who should show no difference in matching native and non-native AV speech, which is still the case at 6 months of age [9], and infants who still prefer to look at a moving mouth rather than a speaker’s eyes, which seems to be before 12 months of age [12].

If the preference for AV native speech (relative to AV non-native speech) increases with age as hypothesized here, this should be reflected by a higher proportion of time spent looking at the English visual speech stream when hearing English, than at the Spanish visual stream when hearing Spanish. However, such a data pattern could also be observed when infants are simply better at detecting AV congruency for native speech than for non-native speech. Although the temporal correlation between sight and sound (including rhythmic cues) is perfect in all AV speech irrespective of language, phonetic AV congruence (including spectral and energetic features that correspond to specific phoneme-viseme pairs) may be detected more easily or in more detail for native speech than non-native speech. This could in turn result in better AV matching performance for native than non-native speech in a preferential looking task. Therefore, to establish AV congruency detection for native versus non-native AV speech, we conducted a second experiment in which infants saw one visual speech story per trial (either in the native or non-native language) while hearing the corresponding auditory story in either the same language (an AV match) or in the other language (an AV mismatch). If perception of AV congruency in native speech is significantly affected by particular cross-modal cues that are not available, or are less prominent, in non-native speech, it is likely to affect infants’ looking behavior in Experiment 2, and offer additional insights regarding the hypothesized pattern of results for Experiment 1.

## Experiment 1

### Methods

#### Ethics statement

The study (that encompasses both Experiments) was approved by the University of Connecticut's Institutional Review Board and was conducted in accordance with the Declaration of Helsinki. Parents gave their informed consent, and were free to leave at any time.

#### Participants

Eighteen full-term infants (9 males) between 154 and 296 days of age (i.e., in between 5 and 10 months old) participated. All infants came from English speaking homes in which there was no exposure to Spanish. Infants were identified and recruited via information packets and request letters sent to individuals associated with birth announcements in the Storrs area (CT, USA). Effects of age were assessed by correlation analyses as well as by assigning infants to one of two age groups (see also result section below) based on a median split of age in days. Given our continuous age sampling, we labeled the resulting two groups as “younger infants” and “older infants”. On average, younger infants were 190 days old (N = 9; SD = 25), and older infants were 265 days old on average (N = 9; SD = 28).

#### Materials

A female simultaneous balanced bilingual (who had learned English and Spanish from birth) was recorded while producing eight stories, each in English and in Spanish. Average story length was 14.5 seconds. The videos were edited in Adobe Premiere Pro to show only the lower half of the speaker’s face (including the lower half of the nose to just below the chin). Videos of the speaker producing a single story in each of the two languages were paired for simultaneous onset. The audio corresponding to one of the two videos was paired with the visual presentation, such that the audio and one of the two videos formed an audiovisual match. The non-matching video was edited to end at the same time as the matching video.

#### Procedure

**Fig 1:**
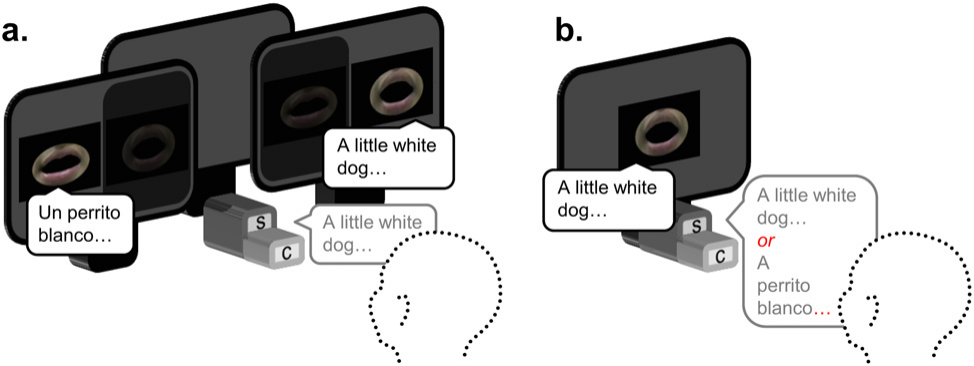
Overview of the set-up for experiment 1 (a.) and 2 (b.). The location of the speaker and camera that recorded looking behavior are indicated by 'S', and 'C' respectively. In the dual-screen set-up in (a.), both visual speech streams were displayed on both screens (to ensure accurate timing) and one stream on each screen was masked by a dark piece of cardboard. The areas inside and surrounding the mouth are erased for display purposes only (to guarantee our actor's anonymity).

Each infant sat on a caretaker’s lap throughout the experiment. Infants’ faces were video recorded for later eye-gaze coding. Three monitors and one central speaker were arranged for stimulus presentation, with the left and right monitors each displaying one of the two visual conditions (e.g., visual Spanish story on left monitor, visual English story on right monitor; see Fig 1). Prior to each trial, an experimenter monitoring the infant from another room activated an attention-getter on the middle monitor that was composed of a colorful geometric shape paired with an interesting sound to return the infant’s attention to midline for the subsequent trial. Each child was presented with two blocks of videos, each composed of the eight visual stories, counterbalanced for side of match, language of the corresponding auditory stimulus, and story order.

## Results

Infants' videos were analyzed frame-by-frame for looking behavior (i.e., an infant looked to the left, to the right, or did not look at the screens) by two naïve coders. Inter-rater reliability was greater than 90% for all videos, and for instances where the two coders were in disagreement, a third coder resolved discrepancies.

We calculated looking times by converting the number of frames during which infants looked at the screen into seconds (1 frame = 33.37 ms), thereby collapsing the data across trials, stories, infant gender and location of the screen (left/right) that displayed the visual speech stream that matched the audio. Next, we calculated the proportion of time infants spent looking at the screens, by dividing the time spent looking at both screens by the total trial times. The overall proportion of time spent looking (.62) was above chance, *t*(17) = 3.19, *p* < .01, indicating that infants were engaged in the task.

**Fig 2:**
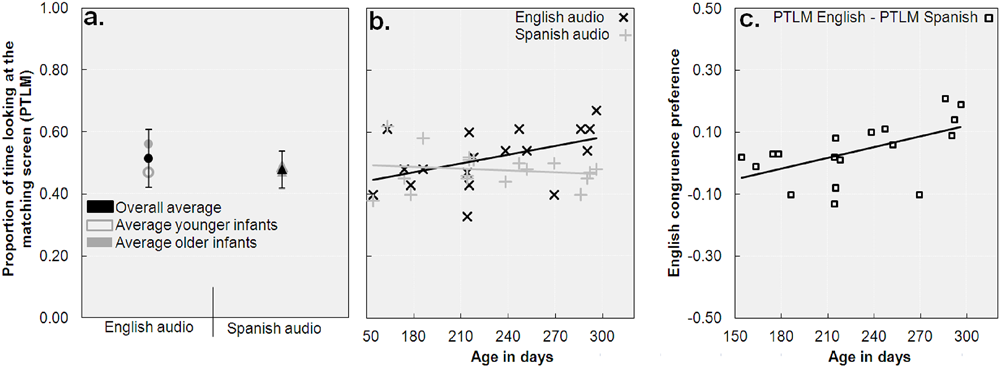
Proportions of time spent looking at the matching screen (PTLM) and congruence preference. Panel a. depicts the average PTLM values. Error bars reflect one standard deviation from the mean. Panel b. shows the individual PTLM values for each language, and panel c. shows the preference for English congruent stimuli over Spanish congruent stimuli (PTLM English—PTLM Spanish).

Next, we calculated the average proportions of time infants looked at the screen that matched the audio (i.e., Proportion of Time Looking at the Match, henceforth PTLM)—separately for English and Spanish audio—by dividing the time spent looking at the screen that displayed the visual speech stream that matched the audio by the time spent looking at both screens (see Fig 2 for averages). The PTLM values within each age group (we applied a median split, see Methods section above for details) were normally distributed (Shapiro-Wilk tests yielded *p*s > .28), and effects of age were therefore analyzed using a 2 (Language; English vs. Spanish) × 2 (Age group; younger vs. older infants) mixed effects ANOVA.

The ANOVA revealed no main effect of Language, *F*(1,16) = 3.23, *p* = .09, *η* ^2^_p_ = .17, indicating that PTLM values were alike for English and Spanish (.51 vs. .48). PTLM values were statistically alike across Age groups, *F*(1,16) = 1.75, *p* = .20, *η* ^2^_p_ = .10, but there was an interaction between Age and Language, *F*(1,16) = 7.36, *p* = .02, *η* ^2^_p_ = .32. As can be seen in Fig 2, this interaction indicated that PTLM values for English and Spanish were alike for younger infants, *t*(8) = .76, *p* = .47, whereas older infants' PTLM was higher for English than for Spanish, *t*(8) = 2.83, *p* = .02. Between group comparisons revealed that older infants' PTLM values for English were higher than for younger infants, *t*(16) = 2.30, *p* = .04, whereas PTLM values for Spanish were alike for older and younger infants, *t*(16) = .53, *p* = .60.

Non-parametric analyses confirmed these results as there was no overall difference in English versus Spanish PTLM values (assessed through a Wilcoxon Signed rank tests, *Z* < 1.51, *p* > .13), whereas the difference was significant for older infants, *Z* = 2.07, *p* = .04, but not for younger infants, *Z* = .30, *p* = .77. Likewise, between group comparisons (Mann-Whitney U tests) confirmed that older infants' PTLM values for English were higher than for younger infants, *U* = 16, *p* = .03, with no such difference for Spanish, *U* = 37, *p* = .80.

Indeed, as age increased, PTLM values for English increased, *r*(16) = .47, *p* = .05, whereas the decrease in Spanish PTLM values was not significant, *r*(16) = -.15, *p* = .56. Moreover, infants' preference for English congruent stimuli over Spanish congruent stimuli increased with age as anticipated, *r*(16) = .54, *p* = .02 (see Fig 2c). Non-parametric analyses confirmed the pattern: *ρ* and *τ*_B_ values for age × PTLM for English were > .38, *p*s < .03, whereas PTLM values for Spanish did not correlate with age, *p* > .79. Similarly, infants' preference for English congruent stimuli over Spanish congruent stimuli was again found to increase with age: *ρ* and *τ*_B_ values > .44, *p*s < .02.

## Experiment 2

### Methods

#### Participants

Eighteen full-term infants (8 males) in between 171 and 308 days of age (i.e., in between 5 and 10 months old) participated. All infants came from English speaking homes where they had no exposure to Spanish. Infants were identified and recruited via information packets and request letters sent to individuals associated with birth announcements in the Storrs area (CT, USA). Infants were assigned to one of two age groups (see also result section below) based on a median split of age in days. On average, the younger infants were 198 days old (N = 9; SD = 16), whereas the mean age in days for older infants was 260 (N = 9; SD = 26).

#### Materials

The same videos used in Experiment 1 were used again in Experiment 2. However, in Experiment 2 infants were presented with a single video at a time in which the female was speaking either English or Spanish. These videos were paired with either the corresponding audio or with audio from the story produced by the same woman in the other language.

#### Procedure

As in Experiment 1, infants sat on a caretaker’s lap and were video recorded throughout the experiment for later eye-gaze coding. In contrast to Experiment 1, however, in this case infants faced a single monitor on which visual trials were presented sequentially with either matching or mismatching audio. Prior to each trial, an attention-getter coupled with an engaging sound appeared on the screen to attract the infant’s attention to midline prior to the onset of the subsequent trial. Each child was presented with eight trials, four in which the audio and visual streams were congruent and four in which they were incongruent. Half of the trials were in Spanish and half in English.

## Results

Infants' videos were analyzed frame-by-frame for looking behavior (i.e., an infant looked towards the single screen or looked away) by two naïve coders. Inter-rater reliability was greater than 90% for all videos, and for instances where the two coders were in disagreement, a third coder resolved discrepancies.

**Fig 3:**
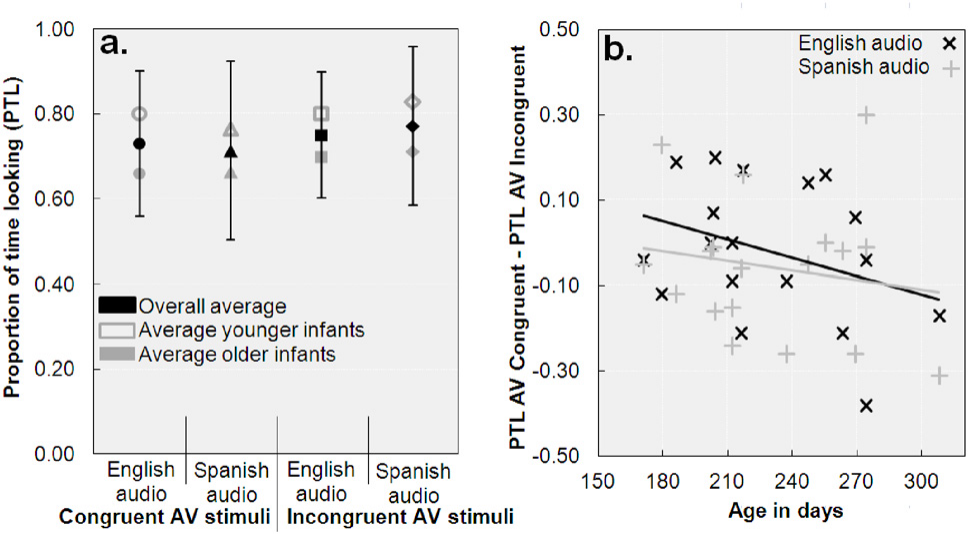
Proportions of time spent looking at the screen (PTL) and congruence preference. Panel a. depicts the average PTL values. Error bars reflect one standard deviation from the mean. Panel b. shows the individual congruence preference scores (PTL for congruent stimuli—PTL for incongruent stimuli), separately for English and Spanish audio.

We calculated the proportion of looking times to the English and Spanish visual speech streams for English and Spanish audio, thereby collapsing the data across trials, stories, and infant gender (i.e., four PTL values, for Proportion of Time Looking), by converting the number of frames infants looked at the screen into seconds (1 frame = 33.37 ms), and dividing the time spent looking at the screen during a trial by the total trial time. The overall PTL (i.e., .75) was well above chance, *t*(17) = 7.01, *p* < .01, as were the individual PTL values for Spanish and English audio combined with Spanish and English visual speech, *t*s(17) > 4.33, *p*s < .01 (see Fig 3 for averages), indicating that infants looked at the screen as intended. Next, we median-split the data into two age groups (see Methods section above for details). Because the distribution of older infants' PTL values for AV congruent trials deviated from normality (Shapiro-Wilk tests yielded *p*s < .04), we analyzed the PTL values using non-parametric methods. None of the Mann-Whitney U tests that tested differences between younger and older infants on the four PTL values reached significance, *U*s > 20, *p*s > .08, indicating that PTL values were alike for younger and older infants (see Fig 3). In each age group, we assessed the differences between the PTL values using Wilcoxon Signed rank tests, and again, none of the comparisons reached significance, *Z*s < 1.72, *p*s > .08. The pattern of results was confirmed by a 2 (Audio; English vs. Spanish) × 2 (Visual speech stream; English vs. Spanish) × 2 (Age; younger vs. older infants) ANOVA (all *p*s > .10).

To gain more insight into the effect of age on AV congruency preference, we calculated individual congruence preference scores by subtracting the PTL for incongruent trials from congruent ones. A positive score thus reflects that infants looked longer at congruent than incongruent trials, whereas a negative score reflects the opposite. As can be seen in Fig 3, there was an overall tendency to look relatively less at the congruent trials as age increased, but this overall trend was not significant, *ρ*(16) = -.26, *p* = .31, nor were the trends for English audio, *ρ*(16) = -.31, *p* = .21, Spanish audio, *ρ*(16) = -.08, *p* = .74, or the difference between English and Spanish, *ρ*(16) = -.10, *p* = .69 (absolute *τ*_B_ values < .24, *p*s > .18).

## Discussion

We hypothesized that a preference for native AV speech over non-native speech would be characterized by an age-related increase in looking at the native visual speech stream when native speech is heard, relative to the non-native visual speech stream when native speech is heard. Indeed, Experiment 1 showed that, for the older group of infants, proportion of looking towards English visual speech when it matched the sound was greater than was proportion of looking towards Spanish visual speech when it matched the sound. This finding was corroborated by the correlation between age and the PTLM difference between languages. Although these findings align with a perceptual narrowing account in which infants are proposed to retain perceptual sensitivity to frequently encountered native stimuli and stimulus properties while losing sensitivity for infrequent and non-native stimuli or stimulus properties [4, 8, 9, 13, 18, 19], we certainly have not obtained direct evidence for perceptual narrowing as we observed no (absence of) discrimination between the languages. However, we did observe a clear preference for native AV speech that became manifest through a higher proportion of time spent looking at the native AV match, consistent with the general direction of successful AV matching as is often observed in preferential looking paradigms [10, 14, 15, 16, 18]. Overall PTLM values were at chance level, but this can be accounted for by the variability and complexity of the stimuli across trials as compared to repeated presentations of relatively simple single-items [10, 14–17], and are not uncommon when using fluent speech [9].

The results of Experiment 2 indicated that the age-related increase in native language preference was probably not driven by a difference in AV congruence preference between the native and non-native language, as we observed no difference in looking times towards the English and Spanish congruent and incongruent visual speech streams. As noted, in both congruent AV presentations, the temporal correlation between auditory and visual speech was perfect (irrespective of whether the stimuli were in the native or non-native language), and similarly, in the incongruent AV stimuli the temporal correlation was poor. Given that temporal alignment across the senses is one of the most prominent factors underlying cross-modal binding [20], it is logical to propose that the temporal correlation between sight and sound provides an important cross-modal perceptual cue for infants as well as adults [9, 10, 21]. This is underscored by a large body of research that has consistently shown substantial effects of presenting AV speech out of synchrony [22, 23–30]. It may therefore not be surprising that we observed no difference in AV congruence detection for native and non-native speech. However, there are many more cues in the AV speech signal that infants may use to detect AV congruence, such as phonetic correspondence between phonemes and visemes. Although it may be that infants perceived more phonetic detail in the native AV congruent material (because Spanish has phonemes that do not exist in English), the use of phonetic information is certainly not mandatory for 4-month-olds [31] and the actual benefit of phonetic cues seems to only become apparent at around six years of age [32, 33] and develop further into adulthood [34]. Given that neither the phonetic cues nor any other cues in the native language (e.g. rhythm, spectral detail, energetic modulations) resulted in differential congruency detection across languages, we suggest that the results in Experiment 1 most likely reflect a genuine preference for native AV speech that increased with age and is not related to differential sensitivity to AV congruency across the two languages.

If anything, Experiment 2 showed that the AV congruence preference was variable across infants. Some infants looked longer at congruent material, which was indicated by a positive PTL difference score (Fig 3b), whereas some preferred to look at incongruent material. Although we do not wish to claim that our data present evidence for a bimodal distribution in terms of (in)congruence preference, it has been suggested that infants prefer AV congruency for speech stimuli that are heard as native-like, and respond to AV incongruency when they detect that the speech is non-native [35]. Perhaps our observed differences in AV congruency preferences were related to a varying ability to distinguish between the languages, but this hypothesis clearly requires further testing.

More importantly, however, is that whenever individual differences in infant data are presented and/or analyzed [10, 13, 36], variability across infants is quite large. As an example of the potential importance of such differences, it was recently demonstrated that in 6- to 9-month-old infants, the individual amplitude of their electrophysiologically-based mismatch response to an AV phonetic incongruency is negatively associated with looking behavior [37]. Because the mismatch response is usually found in younger infants (2- to 5.5-month-olds) [38, 39], Kushnerenko et al. [37] related the individual differences to variability in maturation of multisensory processing. According to this view, the data pattern in Experiment 2 here may reflect individual differences in maturation of the perceptual systems and thus sensitivity to the temporal congruence of AV speech, although this suggestion is clearly speculative at this point.

As argued, Experiment 2 was designed to determine whether an enhanced ability to detect AV congruence in native AV speech could have contributed to the native language preference observed in Experiment 1. Even if AV congruence detection is in fact easier for native speech, it does not provide the leverage needed to produce the clear effects of native speech preference observed in Experiment 1.

Having said that, it is essential to recognize that both experiments are quite different despite involving the same basic stimuli. In Experiment 1, infants were always provided with a matching visual speech stimulus to which the non-match could be compared. This may have required or provoked a different looking strategy than when only one visual speech stream was presented, as in Experiment 2. Given that Experiment 2 is the more likely real-life scenario (i.e., in life, infants are never presented with two simultaneous talking faces while hearing only one voice) and infants effectively use lip-read input to learn phonemes [40], they may have been looking at the screen irrespective of whether unimodal signals were congruent or not (and regardless of the language they heard) simply to try and detect commonalities between sight and sound.

To conclude, we demonstrated that the preference for native AV speech increased in the second half of the first year of life, which seems unlikely to be driven by a variable preference for AV congruence across languages.
